# KDM5c Promotes Colon Cancer Cell Proliferation Through the FBXW7-c-Jun Regulatory Axis

**DOI:** 10.3389/fonc.2020.535449

**Published:** 2020-09-16

**Authors:** Haishan Lin, Nina Ma, Lei Zhao, Guowei Yang, Bangwei Cao

**Affiliations:** ^1^Cancer Center, Beijing Friendship Hospital, Capital Medical University, Beijing, China; ^2^Department of Tropical Medicine, Beijing Friendship Hospital, Capital Medical University, Beijing, China

**Keywords:** KDM5c, c-Jun oncogene, colon cancer cell, FBXW7, epigenetic modifier

## Abstract

KDM5c is a histone demethylase that specifically demethylates trimethylated and dimethylated H3 Lys-4 to play a central role in transcriptional repression. C-Jun is a proto-oncogene and promotes cell proliferation when ectopically accumulated, but can be ubiquitinated by SCF (FBXW7), leading to its degradation. FBXW7 is an E3 ubiquitin ligase of c-Jun, and exhibits carcinostasis in colon cancer. Here, we report that overexpression of KDM5c in human colon cancer cells results in attenuated *FBXW7* transcription and accumulated c-Jun protein, leading to increased proliferation of colon cancer cells. We show that overexpression of KDM5c can result in increased c-Jun protein levels and decreased ubiquitin levels, with no significant change in mRNA levels of *c-Jun*. KDM5c overexpression blocks the ubiquitin-proteasome proteolytic pathway of c-Jun by down-regulating the expression of *FBXW7*. KDM5c down-regulation of *FBXW7* occurs by demethylation of H3K4me3 at TSS and downstream of the *FBXW7* gene. And interaction of KDM5c with H3K4me3 downstream of *FBXW7* gene may be followed by recruitment of DNMT3b to methylate the spatially close CpG island located near the *FBXW7* TSS. This methylation represses *FBXW7* gene expression, which can reduce c-Jun degradation via the ubiquitin-proteasome pathway. TCGA database analysis revealed high expression of KDM5c in colon cancer tissues. KDM5c expression in colon cancer was correlated with poor overall survival of patients in the first 7 years. Data from TCGA showed that high expression of KDM5c was correlated with high DNA methylation of the *FBXW7* gene, but was not positively correlated with methylation of the Jun gene. These results suggest that KDM5c regulation of colon cell proliferation is mainly mediated by the KDM5c-FBXW7-c-Jun axis.

## Introduction

Colorectal cancer (CRC) is the third most prevalent malignancy and ranks second in mortality among solid tumors, representing a serious public health problem worldwide (1). Driver gene mutations account for a large proportion of colon malignant tumors cases, but mutated driver genes have not been identified for some colon cancer cases, suggesting that epigenetic changes act as an important supplement to genetic changes to cause tumors. During tumor development, abnormal epigenetic regulation can aggravate tumor proliferation and metastasis (2–4). Here, we applied an epigenetic perspective to investigate new potential therapeutic targets for colorectal cancer.

Recent evidence has indicated that epigenetic mutations are strongly involved in cancer initiation and progression (5). These mutations have been detected widely across the genome, and are considered a more important contributor to tumor heterogeneity. Many epigenetic modifications play critical role in CRC including DNA methylation, histone modification, chromatin remodeling, microsatellite instability, and non-coding RNAs (6, 7). Thus, epigenetic mutations are promising targets for not only epidemiological and physiopathological studies, but also therapeutic response evaluation and drug design (8).

A member of the SMCY homolog family, KDM5c (also known as JARID1C) is an H3K4me2/3 demethylase that plays a central role in transcriptional repression (9). KDM5c was initially found to be important for brain development and function, and mutations of KDM5c can lead to X-linked mental retardation (10). KDM5c abnormality was subsequently correlated with development of various cancers. For example, KDM5c was significantly upregulated in breast cancer tissues compared with paired normal breast tissues, and was positively correlated with metastasis (11); loss of KDM5c results in the activation of a set of enhancers in human breast cancer cells (12); KDM5c acts in proliferation and invasion of gastric cancer cells, which may be partly associated with p53 expression (13); and KDM5 demethylase suppresses STING-induced innate immune response in tumor cells (14, 15). Despite these associations with various cancers types, the function of KDM5c in CRC progression has not been reported.

*C-Jun* is a proto-oncogene that accelerates cell proliferation (16). It is required for cell cycle progression through the G1 phase, and increased G1 arrest is detected in *c-Jun* null cells (17). The c-Jun protein is ubiquitinated by FBXW7 (F-box/WD repeat-containing protein 7), which belongs to the F-box protein family and functions as a receptor subunit for SCF (Skp1/Cullin/F-box protein) E3 ubiquitin ligases (18, 19). FBXW7 acts as an important tumor suppressor, and mutations in the *FBXW7* gene have been found in ovarian, lymphoma, and colorectal cancers (20, 21). However, whether FBXW7-mediated degradation of c-Jun is under strict control remains unknown.

In this study, we investigated the function and mechanism of KDM5c in colon cancer cell proliferation by disrupting KDM5c expression. Our results showed that KDM5c accelerated proliferation of colon cancer cells by down-regulating *FBXW7* transcription, thereby, reducing c-Jun degradation via the ubiquitin-proteasome pathway. We also observed regulation by KDM5c on *FBXW7* that may be mediated by binding of KDM5c to TSS and downstream of the *FBXW7* gene. We identified a DNA methylation site upstream of the *FBXW7* gene. And the interaction site of KDM5c and H3K4me3 in downstream of *FBXW7* gene may be spatially adjacent and interlinked by DNMT3/DNMT3L. Our study reveals novel roles of KDM5c in regulating colon cancer cell proliferation and suggests KDM5c as an attractive target for CRC treatment.

## Materials and Methods

### Cell Culture

The human colon cancer cell lines RKO and HCT-8, containing the wild-type *FBXW7* gene, were cultured in Dulbecco’s Modified Eagle Medium (DMEM) supplemented with 10% FBS, 1% penicillin-streptomycin, MEM vitamins (Media Tech), and MEM non-essential amino acids (Media Tech). Cells were grown on tissue culture-treated plates (Laboratory Product Sale) in a 37°C humidified incubator and an air atmosphere with 5% CO2. All cell lines were purchased from ATCC and routinely tested and authenticated via by assessing the cell morphology, proliferation rate, a panel of genetic markers, and checking for contamination. Cells were also tested for mycoplasma using the MycoAlert Detection Kit (Cambrex). Human interleukin 4 (IL4) and neutralizing IL4 antibody were purchased from Cell Signaling.

### Transient Transfection

RKO and HCT-8 cells were transiently transfected with 30 nM KDM5c or a scrambled control siRNA using Lipofectamine 3000 (Thermo Fisher Scientific) following the manufacturer’s protocol. For plasmid transfection, 3 μg plasmid was transfected using MSCV-dGFP-JARID1C (Promega) or MSCV-null (Mirus Bio). The cells were used for experiments 48 h after transfection, or as otherwise indicated.

### Cell Proliferation Assay

RKO and HCT-8 cells were seeded in 96-well plates at a density of 2000 cells/well and allowed to attach for 8 h, and 0.5 mg/mL MTT (Sigma-Aldrich) was then added before another incubation of 4 h at 37°C. The violet MTT formazan precipitates were subsequently dissolved in 100 μL DMSO. Absorbance was measured at 570 and 670 nm and adjusted for background using a UQuant reader. The MTT assay was repeated at the same time on four consecutive days. For each group, five replicates and three independent experiments were performed.

### Colony Formation Assay

For the colony formation assay, 1000 RKO or HCT-8 cells were seeded in 6-well plates and incubated overnight. The cells were then transfected with siRNA or mimic plasmid for 24 h. The medium was replaced with fresh medium every 3 days. When visible colonies formed, they were fixed with 4% paraformaldehyde and stained with 0.1% crystal violet. The numbers of colonies were counted using a Syngene G:BOX imaging system.

### Flow Cytometry

Cell cycle progression was analyzed 48 h after transfection using propidium iodide (PI) as a stain to label the DNA content. The isolated cell pellet was washed twice with PBS supplemented with 1% FBS and resuspended in 70% cold ethanol for overnight fixation. The samples were then centrifuged for 10 min at 500 rcf at 4°C, and resuspended in cold PBS. Finally, the samples were centrifuged for 5 min at 300 rcf and 4°C, and then resuspended in DNA staining solution [0.1% Triton X-100 (Sigma Aldrich), 2% propidium iodide (Sigma Aldrich) in PBS]. Samples were then incubated for 30 min at room temperature, and flow cytometric analysis was performed on a BD FACSCalibur device (BD Biosciences). Flow cytometry was also used to confirm cell models were successfully established, with more than 90% of cells synchronized at each stage.

### Immunofluorescence

Immunofluorescence was performed following the standard procedure. Briefly, RKO and HCT-8 cells were seeded on a 6-well plate 48 h after transfection. The cells were then fixed with 4% paraformaldehyde and permeabilized with 0.25% Triton X-100, blocked with 1% BSA in PBST, and probed with primary antibodies against c-Jun (Abcam). Alexa Fluor 488 tagged secondary antibody (Cell Signaling) was used for detection, and the nuclei were stained with DAPI (Sigma) and imaged by a Leica SP8 confocal microscope.

### Chromatin Immunoprecipitation-Quantitative PCR (ChIP-qPCR)

Chromatin immunoprecipitation was performed to analyze the enrichment of select regions and confirm H3K4me3 binding at regions of interest using a True MicroChIP Kit (Diagenode) according to the manufacturer’s protocol. A total of 1×10^6^ cells were collected and crosslinked with 1% formaldehyde. Cells were disrupted by ultrasonication to fragment the DNA into 200–500 bp pieces. Specific antibodies to the protein of interest [anti-KDM5c (Abcam) and anti-H3K4me3 (Diagenode)] were added to bind target protein-DNA complexes, and incubated overnight. Protein A agarose was added to bind the antibody-target protein-DNA complexes, washed to remove non-specific binding, and then the enriched target protein-DNA complexes were eluted from the beads and the crosslinks were reversed. After purification, enriched DNA-fragments were subjected to qPCR analysis using fluorescence quantitative PCR. Goat IgG was used as the negative control. The fold change in the amount of the DNA fragment enriched by a specific antibody versus the total input was calculated by the following formula: % recovery = 100^∗^2^∧^[(Ct(input)-log%(x%)/log2)-Ct(sample)]. The primers used in the ChIP-PCR assays are listed in the Table 1.

**TABLE 1:** The primers used in the ChIP-PCR assays.

Location	Primers
1# FBXW7 TSS (chromosome 4 152318937) F	5′-AGGTCCCAACAAGCATCAGA-3′
1# FBXW7 TSS (chromosome 4 152318937) R	5′-CCAGCTTTGTGTTTGAGGCT-3′
2# FBXW7 TSS (chromosome 4 152319177) F	5′-GGTGCTGGACTTTGATGTGG-3′
2# FBXW7 TSS (chromosome 4 152319177) R	5′-AACATCCTGCACCACTGAGA-3′
3# FBXW7 TSS (chromosome 4 152319971) F	5′-ACTCCCAGTGGCCAAACTTA-3′
3# FBXW7 TSS (chromosome 4 152319971) R	5′-GGCTCAAGTTTCAGTGGCAA-3′
4# FBXW7 TSS (chromosome 4 152320116) F	5′-TTGCCACTGAAACTTGAGCC-3′
4# FBXW7 TSS (chromosome 4 152320116) R	5′-TCTCCACAGAACAGGCAAGT-3′
5# FBXW7 (chromosome 4 152534873) F	5′-ACGTTTGTACTCAAGCCGCA-3′
5# FBXW7 (chromosome 4 152534873) F	5′-TTGGATAACGTGTGGTCGGG-3′
6# FBXW7 (chromosome 4 152535061) F	5′-GACCACACGTTATCCAACGC-3′
6# FBXW7 (chromosome 4 152535061) R	5′-CATTTGGCCCCAAACAGACC-3′
7# FBXW7 (chromosome 4 152535162) F	5′-GATCAGTCCGGCTTTTCGAG-3′
7# FBXW7 (chromosome 4 152535162) R	5′-GATCTTACCCCTGACCCGAG-3′
8# FBXW7 (chromosome 4 152539858) F	5′-CCACCATTCCCCTGTTGTAAGA-3′
8# FBXW7 (chromosome 4 152539858) R	5′-GACCTGAAGTTCCAAGAGCCA-3′
9# FBXW7 (chromosome 4 152540657) F	5′-TCTCGAAAGCTCCAAACCGT-3′
9# FBXW7 (chromosome 4 152540657) R	5′-TCCTCGCGCAGATTGTTAGG-3′
1# c-Jun TSS (chromosome 1 58778991) F	5′-GCAATGAACCCAAGGCTGAA-3′
1# c-Jun TSS (chromosome 1 58778991) R	5′-TCCTGTGAGAAGCATCGAGG-3′
2# c-Jun TSS (chromosome 1 58779638) F	5′-GCGTGACTTTATGCGAGTGT-3′
2# c-Jun TSS (chromosome 1 58779638) R	5′-CCGGTGTTAGTCTACTCCCC-3′
3# c-Jun TSS (chromosome 1 58780100) F	5′-GGAGACCGCCCCTAAACTTA-3′
3# c-Jun TSS (chromosome 1 58780100) R	5′-GAGGGGTGGTTGTTGTTTCC-3′
4# c-Jun (chromosome 1 58784487) F	5′-AACCTCAGCTCTGGGGAAATG-3′
4# c-Jun (chromosome 1 58784487) R	5′-CTGCTAATGAGCAAACAGCCC-3′
5# c-Jun (chromosome 1 58784705) F	5′-GTACCCAGTAGGTCTGGGAGT-3′
5# c-Jun (chromosome 1 58784705) R	5′-CCTTCCGGGTTGCTGACATC-3′
6# c-Jun (chromosome 1 58785088) F	5′-CACCACTCCCCAGTTTGCTT-3′
6# c-Jun (chromosome 1 58785088) R	5′-ACGATGTGTCACCAGCTTCAT-3′
7# c-Jun (chromosome 1 58785389) F	5′-AGCTGGTGACACATCGTCAT-3′
7# c-Jun (chromosome 1 58785389) R	5′-GAACTCTGGGAGGGTCGAAT-3′

### Chromosome Conformation Capture (3C) Assay

A total of 1×10^6^ cells were collected and crosslinked with 1% formaldehyde. After stopping the crosslinking via glycine, the cells were lysed using lysis buffer and centrifuged to remove cellular debris. The chromatin was then diluted 3-fold using a ChIP dilution buffer containing a protease inhibitor cocktail (Sigma-Aldrich) and digested overnight at 37°C with restriction enzymes including *Eco*NI, *Sna*BI, *Sal*I, and *Not*I (New England Biolabs). The digested chromatin was further diluted 6-fold into a T4 ligation buffer before ligation was performed for 4 h at room temperature using T4 DNA ligase and 0.5 mM ATP. DNA was purified with the QIAquick PCR Purification Kit (Qiagen) followed by CHIP-qPCR. For CHIP assay, anti-DNMT3 (Abcam) was used to bind target protein-DNA complexes. The primers used in the 3C-ChIP-PCR assays are listed in the Table 2.

**TABLE 2:** The primers used in the 3C-ChIP-PCR assays.

Location	Primers
P1 FBXW7 (chromosome 4 152534873) F	5′-ACGTTTGTACTCAAGCCGCA-3′
P1 FBXW7 (chromosome 4 152534873) R	5′-TTGGATAACGTGTGGTCGGG-3′
P2 FBXW7 (chromosome 4 152535162) F	5′-GATCAGTCCGGCTTTTCGAG-3′
P2 FBXW7 (chromosome 4 152535162) R	5′-GATCTTACCCCTGACCCGAG-3′
P3 FBXW7 (chromosome 4 152325958) F	5′-AGGAAACCGCTACAGACCAA-3′
P3 FBXW7 (chromosome 4 152325958) R	5′-GGGAAGAGGAAGTGGGGATC-3′
P4 FBXW7 (chromosome 4 152325311) F	5′-CCAAGACCAGAAGCTCTCGA-3′
P4 FBXW7 (chromosome 4 152325311) R	5′-CTCGCGCAGATTGTTAGGG-3′

### Co-immunoprecipitation IP/Re-IP

Cells from a 10-cm plate were resuspended in lysis buffer supplemented with a protein inhibitor (PI). The lysate was then centrifuged to collect the supernatant, which was further incubated with primary antibodies (e.g., anti-c-Jun) via shaking at 4°C for 24 h. Then, a 50% slurry of Protein A/G Agarose Resin was added and incubated for two more hours. The resin was washed at least four times using a wash buffer with PI. Finally, the binding proteins were eluted using an SDS loading buffer. Re-IP was performed to re-enrich the antigen-antibody complex eluent obtained from IP using antibodies (e.g., anti-c-Jun) and Protein A/G Agarose Resin to reduce interference from non-chemical bond aggregation.

### Assessment of DNA Methylation

Genomic DNA was extracted using a TIANamp Genomic DNA Kit (Tiangen) and treated with sodium bisulfate using an EZ DNA Methylation Kit (Zymo Research) according to the manufacturers’ protocols. Three separate bisulfite (BSP) modification treatments were performed for each DNA sample. BSP primers (Table 3) were designed using the online MethPrimer software and 50 ng of genomic DNA was used for PCR amplification using Zymo Taq Premix (Zymo Research). A standard amplification program was used with annealing for 40 s at 50.4°C and extension for 30 s at 72°C (38 cycles). The PCR products were then sub-cloned into the pMD19-T vector (Takara) and different positive clones for each sample were randomly selected for sequencing (Sangon). Finally, the sequences were analyzed using online QUMA software.

**TABLE 3:** BSP primers for DNA methylation.

Location	DNA methylation Primers
FBXW7 (chromosome 4 152325440) F	5′-AAAAATTTTTTAGTAATTTTTTAGAGG-3′
FBXW7 (chromosome 4 152325440) R	5′-TTAAATACAAAATCACAACCTAAATC-3′
FBXW7 (chromosome 4 152325675) F	5′-GTTTGTATTTTTATTATATTTTTTGAGTT-3′
FBXW7 (chromosome 4 152325675) R	5′-CCCTACAACCTAATCTACACCTACT-3′
FBXW7 (chromosome 4 152325974) F	5′-AGGAGTAGTTTTTATTTGTTTYGAAG-3′
FBXW7 (chromosome 4 152325974) R	5′-TCTATACRAAACTCTCRCCTCACTC-3′
c-Jun (chromosome 1 58779165) F	5′-GGAAAGTATATTTGGTTTTGTTAAA-3′
c-Jun (chromosome 1 58779165) R	5′-TTCATTTCCCTCATCTACAAAT-3′
c-Jun (chromosome 1 58779351) F	5′-TTTGTAGATGAGGGAAATGAAG-3′
c-Jun (chromosome 1 58779351) R	5′-TAAACTTCAAATCTCTACACTCCC-3′
c-Jun (chromosome 1 58779589) F	5′-AGTGTAGAGATTTGAAGTTTAGGTT-3′
c-Jun (chromosome 1 58779589) R	5′-TAACAAAATCCAAATAAAAACAA-3′
c-Jun (chromosome 1 58779949) F	5′-GGTGTAAYGGAGATTTAGTTGA-3′
c-Jun (chromosome 1 58779949) R	5′-TTTCCCCACTTATAAAACCC-3′
c-Jun (chromosome 1 58780320) F	5′-AAAATAATTGGTTAGGTTTTTTGG-3′
c-Jun (chromosome 1 58780320) R	5′-ATAACCCATAATATCACCCCAA-3′

### Western Blot Analysis

Anti-KDM5c (1:1,000), anti-c-Jun (1:1,000), anti-FBXW7 (1:1,000), anti-Cyclin D1 (1:1,000), anti-H3K4me3 (1:1,000), anti-H3 (1:1000), and anti-β-actin (1:4,000) were used for Western blot analysis according to standard procedures.

### Quantitative PCR (qPCR)

Total RNA was extracted using a Qiagen RNeasy Mini Kit (Qiagen) according to the manufacturer’s protocol. A total amount of 2 μg RNA was used for reverse transcription using Superscript II (Invitrogen). Quantitative PCR (qPCR) was performed in triplicate on an ABI Prism 7500 real-time PCR system (Applied Biosystems) using SYBR Green Premix (Takara). GAPDH was used as the internal control. The relative expression of genes was calculated by the 2-(ΔΔCt) method. Primers used for the RT-qPCR assays were designed using PrimerBank (Table 4).

**TABLE 4:** Primers used for the RT-qPCR.

Location	DNA methylation Primers
GAPDH-CDS-F	5′-GGAGCGAGATCCCTCCAAAAT-3′
GAPDH-CDS-R	5′-GGCTGTTGTCATACTTCTCATGG-3′
C-JUNF-CDS-F	5′-TCCAAGTGCCGAAAAAGGAAG-3′
C-JUNR-CDS-R	5′-CGAGTTCTGAGCTTTCAAGGT-3′
FBWX7-CDS-F	5′-GGCCAAAATGATTCCCAGCAA-3′
FBWX7-CDS-R	5′-ACTGGAGTTCGTGACACTGTTA-3′
KDM5c-CDS-F	5′-GGGTCCGACGATTTCCTACC-3′
KDM5c-CDS-R	5′-ATGCCCGATTTCTCTGCGATG-3′
Cyclin D1-CDS-F	5′-GCTGCGAAGTGGAAACCATC-3′
Cyclin D1-CDS-R	5′-CCTCCTTCTGCACACATTTGAA-3′

### Bioinformatics Analysis of the Association Between the KDM5c Expression and FBXW7/c-Jun Methylation Levels in Colon Cancer Patients

The gene expression data and DNA methylation data (BeadChip platform) from 464 samples were downloaded from TCGA website^1^. From these data, we extracted beta-values to evaluate the DNA methylation level of each probe. The annotations of probes to specific genes (e.g., KDM5c and c-Jun) were defined as probes located at the promoter region of genes. We used the “champ.DMP” function in the “ChAMP” package in R to identify differentially methylated probes. We defined probes with adjusted *p*-value ≤ 0.05 as differentially methylated probes. For the gene expression data, the KDM5C high expression group was defined as expression higher than 1.25 times the median expression and the low expression group was defined as expression lower than 0.75 times the median expression of KDM5c.

### Bioinformatics Analysis of the Association Between KDM5c Gene Expression and Overall Survival in Patients With Colon Cancer

The association between the identified KDM5c gene expression and overall survival (OS) for colon cancer patients was assessed using data from TCGA. Kaplan-Meier plots were constructed to illustrate the relationship between gene expression levels of KDM5c and patient overall survival. The relationship was tested by log-rank test.

### Statistical Analyses

Statistical analyses were performed using GraphPad Prism software (version 5.0). One-way ANOVA followed by Newman–Keuls *post hoc* analysis was used to determine differences between KDM5c-OE, empty vector, KDM5c-KD, and siControl. Tumor data were analyzed using a Student’s *t*-test for comparison of two groups (KDM5c-OE and empty vector or KDM5c-KD and siControl). Any statistical data that did not pass the equal-variance test (Bartlett’s test for equal variances) were logarithmically transformed and reanalyzed. The data presented are the mean ± standard error. The overall survival (OS) rates were analyzed via Kaplan-Meier survival analysis with the log-rank test. All data analyses and statistical correlations of TCGA datasets were performed using R software. A value of *p* < 0.05 is considered statistically significant.

## Results

### KDM5c Is Required for Cell Proliferation and Cell Cycle Regulation in Colon Cancer Cells

**FIGURE 1:**
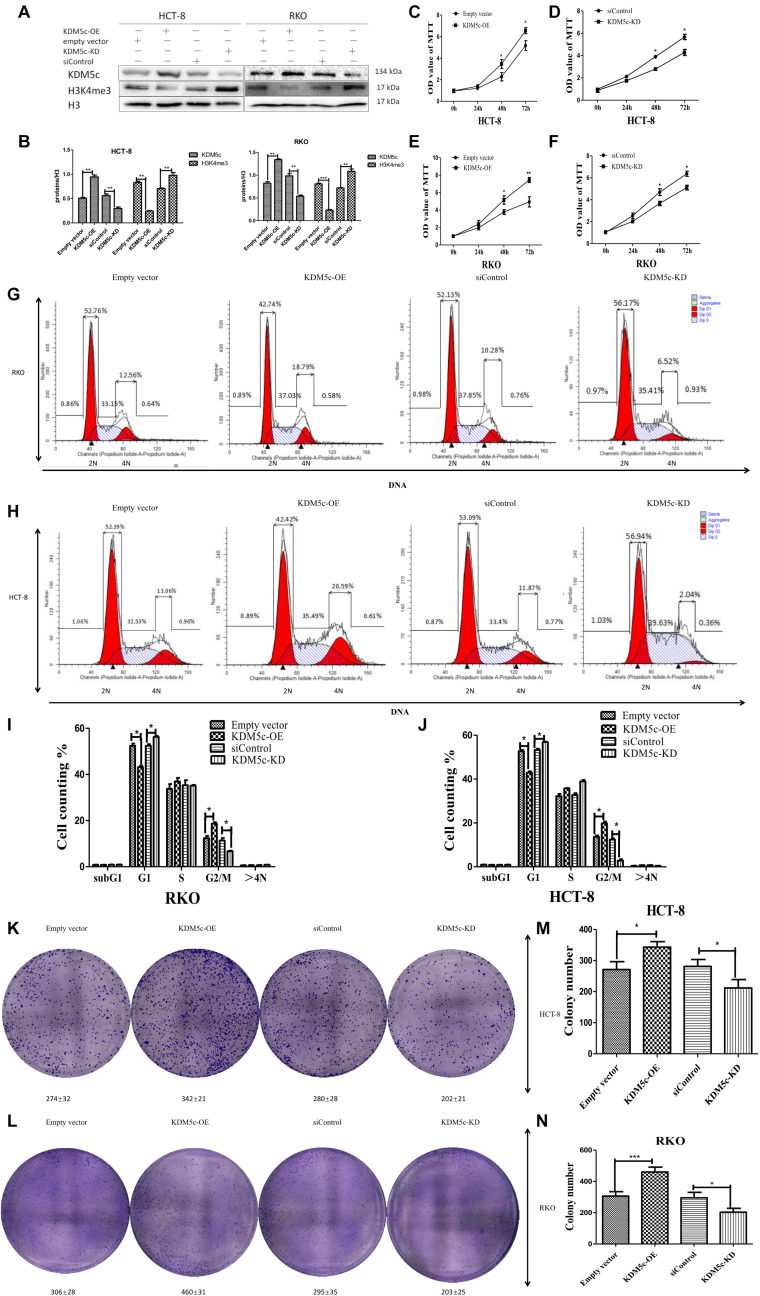
Effects of KDM5c overexpression and knock-down on H3K4me3 levels and colon cancer cell growth. **(A,B)** Representative Western blots **(A)** and quantification **(B)** showing the changes of the H3K4me3 levels in HCT-8 and RKO transfected with the empty vector, KDM5c-OE, siControl, and siKDM5c. H3 was detected as the loading control. **(C–F)** MTT assays to analyze the cell proliferation rates of HCT-8 and RKO 72 h after transfection with the empty vector, KDM5c-OE **(C,E)**, siControl, and siKDM5c **(D,F)**. **(G,H)** Representative flow plots of RKO (upper row) and HCT-8 (lower row) cells transfected with the empty vector, KDM5c-SMCV, siControl, and siKDM5c. Propidium iodide staining and flow cytometric analysis were performed to determine the fractions of G1 and G2/M cells. **(I,J)** The fraction of G2/M phase cells was increased in KDM5c-OE RKO (left) and HCT-8 (right) cells, while the fraction of G1 was decreased in siKDM5c RKO (left) and HCT-8 (right) cells. **(K,L)** Results of 2D colony formation assay showing differences in colony formation in HCT-8 **(K)** and RKO **(L)** cells transfected with the empty vector, KDM5c-OE, siControl, and siKDM5c. **(M,N)** The KDM5c-OE group showed increased colony formation as compared to the empty vector, but siKDM5c decreased colony formation as compared to siControl in HCT-8 **(M)** and RKO **(N)** cells. Each error bar represents the standard error of the mean (SEM). Statistical analysis was performed and *p*-values were calculated. This experiment was repeated three times with similar results. * Above the bars indicates significant difference; *P ≤ 0.05; **P ≤ 0.01; ***P ≤ 0.001.

Previous work has correlated KDM5c to various cancers, but whether KDM5c plays a role in colon cancer progression remains unknown. To answer this question, we first investigated if cell proliferation can be affected by altering expression of KDM5c. To do this, we increased the amount of KDM5c by transfected KDM5c plasmid and used western blot to confirm increased KDM5c protein level in both RKO and HCT-8 cells compared to the wild-type cells, but decreased H3K4me3 protein level, consistent with the demethylase function of KDM5c. Conversely, knockdown (KD) of KDM5c using short siRNAs in RKO/HCT-8 cells (with high endogenous KDM5c levels) reduced KDM5c protein level and increased H3K4me3 protein level (Figures 1A,B), which indicates the successful disruption of KDM5c expression in RKO/HCT-8 cells. Next, MTT assay using these cell lines showed that KDM5c overexpression (OE) significantly promoted HCT-8/RKO cell growth (Figures 1C,E). Consistent with this, KDM5c-KD obviously inhibited cell proliferation compared to the siControl group (Figures 1D,F), suggesting that KDM5c has an important role in colon cancer cell proliferation, which has not been previously reported. In addition, we performed a 2-D colony formation assay in KDM5c-OE HCT-8/RKO cells or KDM5c-KD HCT-8/RKO cells. The overexpression of KDM5c increased colony formation of HCT-8/RKO cells, whereas KDM5c-KD reduced colony formation in HCT-8/RKO cells (Figures 1K,L). These results were statistically significant (Figures 1M,N). To determine if KDM5C regulates the cell cycle, we next performed flow cytometry after cell cycle synchronization. The results showed that more than 90% of the cells were synchronized at each stage, indicating that the cell model was successfully established. In order to directly distinguish the M phase cells, flow cytometry assays were carried out with Propidium Iodide (PI) staining. For KDM5c-OE RKO/HCT-8 cells, there were more cells in G2/M phase and fewer cells in G1 phase and for KDM5c-KD RKO/HCT-8 cells, there were fewer G2/M phase cells and more G1 phase cells (Figures 1G–J), suggesting KDM5c is involved in cell cycle regulation. Together, these results indicate KDM5c is an important determinant of colon cancer cell proliferation and cell cycle.

### KDM5c Promotes c-Jun Protein Accumulation but Downregulates *FBXW7* Expression

**FIGURE 2:**
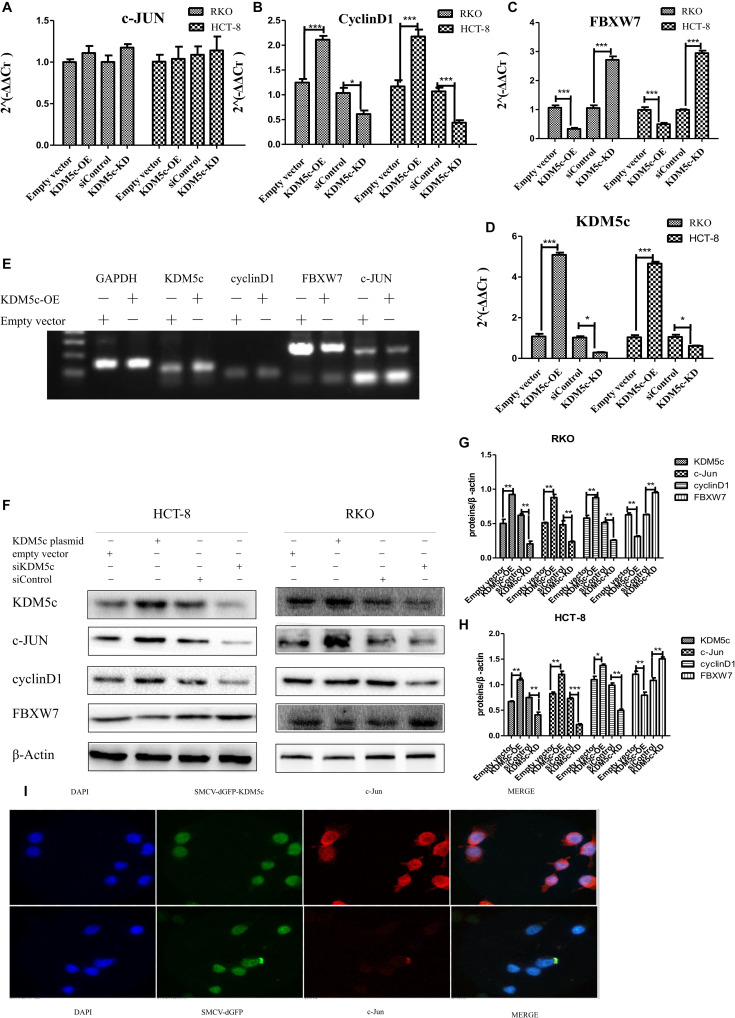
KDM5c overexpression modified downstream gene expression. **(A–D)** Quantitative PCR (qPCR) data of c-Jun **(A)**, Cyclin D1 **(B)**, FBXW7 **(C)**, and KDM5c **(D)** expression in RKO and HCT-8 cells that were transfected with the empty vector (Control), KDM5c-SMCV (KDM5c-OE), siRNA (siControl), and siKDM5c. **(E)** RT-PCR results showing the kinetic transcription levels of KDM5c, cyclin D1, and FBXW7. **(F–H)** Western blot **(F)** and qualification **(G,H)** analysis showing that the overexpression of KDM5c in both RKO and HCT-8 cells increased c-Jun and cyclin D1 but reduced FBXW7 protein levels. The level ofβ-Actin was detected as the loading control. **(I)** Immunofluorescence indicates that HCT-8 cells transfected with MSCV-dGFP-KDM5c expressed more c-Jun than those transfected with MSCV-dGFP-null. The presented data are from two independent experiments. Upper panel: staining of HCT-8 overexpression of KDM5c. Lower panel: cell nucleus (blue), KDM5c (green), and c-Jun (red) staining of HCT-8 transfected with the empty vector. Merge: cell nucleus (blue), KDM5c (green), and c-Jun (red) overlap. Scale bar = 25 μm. Data indicate the mean ± SD from four technical replicates. Non-significant, *P* > 0.05; *P ≤ 0.05; **P ≤ 0.01; ***P ≤ 0.001. This experiment was repeated three times with similar results.

To investigate the mechanism underlying KDM5c regulation of RKO/HCT-8 cell growth, we used quantitative PCR (qPCR) and Western blot methods to examine changes of multiple cancer-related genes at the transcription and protein level when KDM5c expression was altered. Levels of c-Jun, FBXW7, and Cyclin D1 were measured with different amounts of KDM5c expression. When KDM5c was overexpressed, FBXW7 expression was significantly decreased compared to the level in the control group (*P* < 0.05), while in the KDM5c-KD group the FBXW7 mRNA level was much higher than that of the control group (Figure 2C). As shown by Western blot and qPCR results, the FBXW7 protein and RNA levels changed similarly in the different groups tested, indicating that KDM5c downregulates FBXW7 expression. However, there was no difference in the mRNA expression of c-Jun between the two groups (Figure 2A), but the protein level was much higher in KDM5c-OE and lower in the KDM5c-KD group relative to the level in the control. Furthermore, the downstream target of c-Jun, cyclin D1, exhibited the same changes in both mRNA and protein levels as c-Jun and KDM5c (Figures 2A–F). We tested the effects of expression of KDM5c fused to green fluorescence protein (GFP). The immunostaining results also showed increased c-Jun (red) levels in HCT-8 cells overexpressing the GFP-KDM5c fusion protein, with more than 40% of GFP-positive cells showing higher signal for c-Jun (Figure 2I). In contrast, c-Jun levels were quite stable in cells transfected with control vectors. These data indicate KDM5c promotes c-Jun protein accumulation but downregulates FBXW7 expression.

### Co-localization of KDM5c and H3K4me3 in FBXW7

**FIGURE 3:**
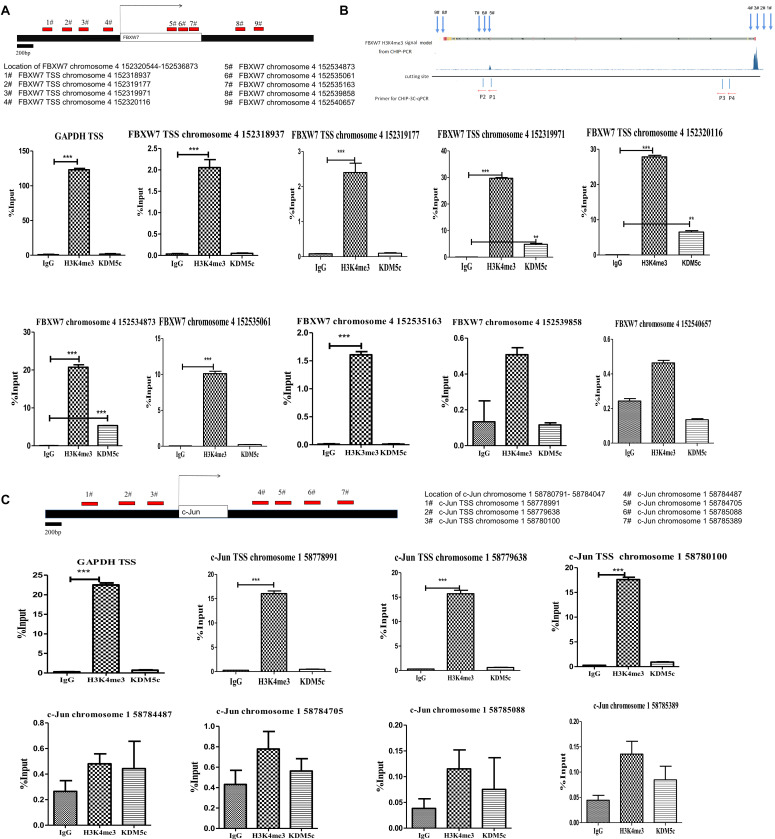
H3K4me3 modification level and its binding proximity to KDM5c as determined by ChIP-qPCR assays. **(A,B)** Primer locations selected according to the H3K4me3 distribution patterns in the FBXW7 gene. The #1–4 primers, respectively, anneal to chromosome 4 152318937, 152319177, 152319971, and 152320116 in the TSS region of FBXW7; the #5–9 primers, respectively, anneal to chromosome 4 152534873, 152535061, 152535163, 152539858, and 152540657 in the downstream of the FBXW7 gene. The H3K4me3 peak distribution was determined by ChIP-qPCR using H3K4me3-specific antibodies. The location of KDM5c reciprocal DNA was determined by KDM5c-specific antibodies. The structure diagram shows the relationship between the loci and the anchor points in 3C-CHIP (showed in Figure 4) **(B)**. The ChIP-qPCR values are shown below. H3K4me3 enrichments were tested in #3–4 and #5–6 locations in FBXW7 gene. Enrichment of KDM5c was detected in the #3–4 and #5 locations in the FBXW7 gene. **(C)** Primer locations chosen according to the H3K4me3 distribution patterns in the c-Jun gene. The #1–3 primers, respectively, anneal to chromosome 1 58778991, 58779638, 58780100 in the TSS region of the c-Jun gene; the #4–7 primers, respectively, anneal to chromosome 1 58784487, 58784705, 58785088, and 58785389 in the downstream of the c-Jun genes. A H3K4me3 enrichment was tested in the #1–3 locations. And no obvious enrichment of KDM5c also was detected in the *c-Jun* gene. Experiments were repeated three times, each with three qPCR measurements, and the value for the representative experiment is shown as the mean ± SEM. * Above the bars indicates significant difference; *P* > 0.05; *P ≤ 0.05; **P ≤ 0.01; ***P ≤ 0.001.

We next asked if KDM5c acts by binding directly to the *FXBW7* or *c-Jun* gene regions to regulate their expression. H3K4 trimethylation (H3K4me3) is a well-known epigenetic modification that promotes mRNA expression, and typically localizes close to the transcription start site (TSS) and up to 5 kb downstream on actively transcribed genes (ENCODE Project Consortium 2007). To do this, we performed ChIP-PCR in RKO cells overexpressing KDM5c to investigate KDM5c association with *FXBW7* and *c-Jun* genes and determine the potential correlation of KDM5c with H3K4me3 signals and transcriptional regulation. We probed the TSS and the surrounding sequence, up to 5 kb downstream (Figures 3A,B). The primers used for ChIP-qPCR assays were selected based on the H3K4me3 binding signal of the FBXW7 ChIP data in the ENCODE database, The primers used for ChIP-qPCR assays were selected based on the H3K4me3 binding signal of the FBXW7 ChIP data in the ENCODE database, which showed an obvious H3K4me3 peak in the TSS region of the *FBXW7* gene. Four primers were in TSS region and five primers downstream of the *FBXW7* gene. To confirm this, ChIP-PCR was performed to look at H3K4me3 binding and consistently with the ENCODE data (Figure 3B), revealed significant enrichments of H3K4me3 peaks in this same region. An obvious KDM5c enrichment was detected in the FBXW7 gene TSS between chromosome 4 152319971 and 152320116. And KDM5c signal was also observed in a region downstream of the FBXW7 transcription area at chromosome 4 152534873. Furthermore, a H3K4me3 peak was also tested in the *c-Jun* gene TSS between chromosome 1 58778991 and 58780100. While, no obvious KDM5c enrichment was teseted in the *c-Jun* gene TSS and within 2 kb of downstream sequence (Figure 3C). These data suggest that KDM5c does not directly control c-Jun expression, but promotes c-Jun accumulation by suppressing FBXW7 expression.

### KDM5c Adjusts DNA Methylation Loci in the FBXW7 Gene Region

**FIGURE 4:**
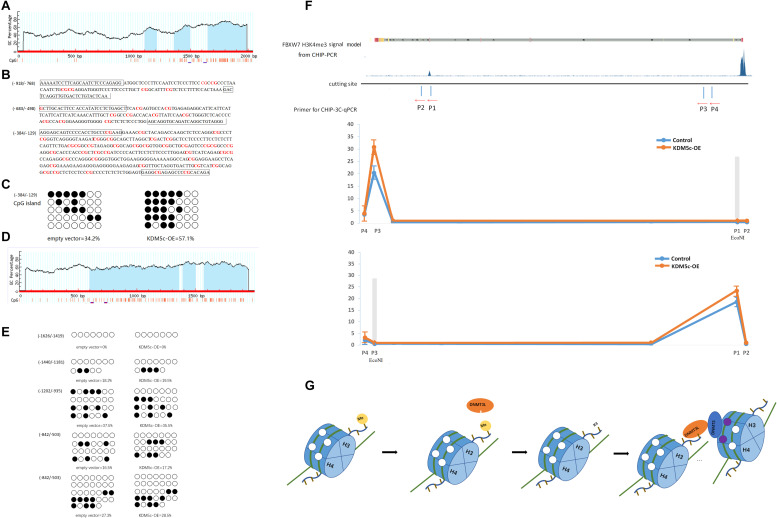
KDM5c overexpression modifies the DNA methylation profile of the FBXW7 gene and its potential regulation. **(A)** Schematic representation of the FBXW7 promoter. The FBXW7 gene is located on chromosome 4, and a 2-kb promoter region spans from 152324318 to 152326358 (NCBI accession NG_029466). The GC percentage is indicated by blue background. The *x*-axis denotes the bp position in the 5′-untranslated region relative to the TSS. **(B)** Sequence of the CpG islands of the FBXW7 gene. The methylation loci are marked in red letters and the primer annealing positions are boxed. **(C)** The percentages of (384/129) CpG islands were analyzed using QUMA software. **(D)** Schematic representation of the c-Jun promoter. The c-Jun gene is located on chromosome 1, and a 2-kb promoter region spans from 58778791 to 58780791 (NCBI accession NC_000001.11). The GC percentage is indicated by. The *x*-axis denotes the bp position in the 5′-untranslated region relative to the TSS. **(E)** The percentages of *c-Jun* CpG islands were analyzed using QUMA software. **(F)** The relative association between the (384/129) CpG islands and the H3K4me3 peak interacting with KDM5c was detected by ChIP-3C-qPCR assays in RKO cells. The upper panel shows the H3K4me3 of the FBXW7 ChIP-seq signal in ENCODE, the *Eco*NI cleavage site, and the locations of primers used for ChIP-3C-qPCR assays. The lower panel shows the qPCR results. **(G)** Schematic figures showing how KDM5c adjusts the methylation of distant CpG islands.

The online software MethPrimer^2^ predicts that chromosome 4 152324318–152326358, which was between TSS and the first exon of the *FBXW7* gene, contains three CpG islands at −918/768, −683/498, and −384/129 (Figure 4A). And TSS of *c-Jun* (chromosome 158778791–58780791) also contains 3 CpG islands at −1420/641, −638/499, and −429/58 (Figure 4D). The sequences of FBXW7 CpG islands are presented in Figure 4B, with the methylation loci indicated in red letters and the primer-annealing positions boxed. The methylation patterns of these CpG sites were determined using bisulfite-assisted sequencing for RKO cells transfected with empty vector or KDM5c-SCMV in three individual trials. The same CpG sites were identified in all clones. The methylation percentages of all CpG sites in the three CpG islands were determined for the *FBXW7* and *c-Jun* RKO empty vector and KDM5c-OE and further assessed using QUMA software^3^. As shown in Figure 4C, the DNA methylation levels of (384/129) CpG island changed significantly between the empty vector and KDM5c-OE (34.2 ± 5.26% and 57.1 ± 6.95%). However, no methylation was detected for the other two CpG islands, (918/768) and (683/498), showed, suggesting that the (384/129) CpG island of the FBXW7 gene plays a major role in regulating the significantly higher DNA methylation levels adjusted by KDM5c. Furthermore, five primers were set up for detection the three CpG islands in the TSS of c-Jun gene (the sequence of CpG islands are presented in Supplementary Material), we found that KDM5c overexpression did not affect the methylation of CpG in *c-Jun* gene (Figure 4E), suggesting that KDM5c does not affect *c-Jun* gene expression by affecting DNA methylation.

### DNMT3b May Mediate the Formation of DNA Looping Between CpG Islands Near the TSS and the H3K4me3 Peak Downstream of FBXW7

H3K4me3 in the TSS region is a recognized transcription initiation marker, which achieves transcriptional inhibition through histone and DNA bimodal methylation (22). However, we want to further explore how downstream H3K4me3 demethylation can achieve transcriptional inhibition. Despite their linear distance from each other, we hypothesized that the target sites of KDM5c and DNA demethylase might be brought physically into close proximity to each other by DNA looping. Based on previous studies that suggested DNMT3B expression might contribute to the CpG island methylator phenotype in colorectal cancer (23), we investigated DNMT3B as a DNA demethylase conformer member. If close together, KDM5c- and DNMT3B-binding fragments digested by the same restriction enzymes should be able to be ligated and detected by qPCR method. To test this hypothesis, we performed ChIP combined with chromosome conformation capture (ChIP-3C) assays in RKO cells. We designed two primers (P3 and P4) located in the promoter region flanking CpG island candidates. The binding site of P3 targets the position of CpG island near the TSS of FBXW7, which is the position where methylation is controlled by KDM5c (Figure 4F). In addition, we designed another two primers (P1 and P2) downstream of FBXW7. P1 binds near the region of DNA bound by KDM5c. *Eco*NI restriction enzyme only acts on the DNA fragment amplified by P3 and P1, *Sna*BI only acts on the DNA fragment amplified by P3 and P2, *Sal*I only acts on the DNA fragment amplified by P4 and P1, and *Not*I only acts on the fragment produced by amplification by P3 and P2. As shown in Figure 4D, after cleaving the gene fragment with *Eco*NI restriction enzyme, the gene fragment enriched with DNMT3B antibody can be amplified by PCR detection using P1 and P3 primers. Thereby, we speculate that KDM5c demethylates H3K4me3 bound downstream of FBXW7 and recruits DNMT3 to remove the methylation of the DNA CpG island located upstream of the FBXW7 TSS, resulting in the downregulation of FBXW7 expression. All in all, KDM5c can increase the exposure of H3K4 by removing H3K4me3 methylation, recruit the DNMT3/DNMT3L complex containing, and induce demethylation of no matter linear distance proximity or spatially adjacent DNA CpG island to induce transcription initiation (Figure 4G).

### KDM5c Overexpression Inhibits c-Jun Degradation via the Ubiquitin-Proteasome Pathway

**FIGURE 5:**
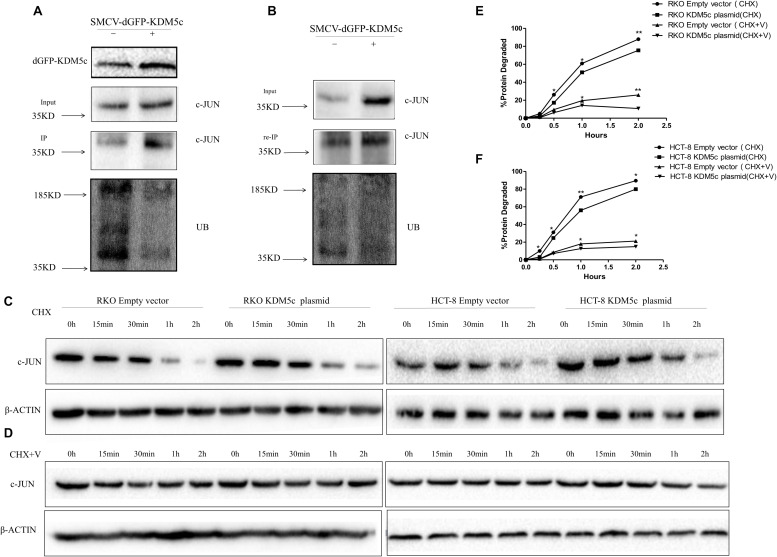
KDM5c reduces proteasome-mediated c-Jun degradation. **(A)** KDM5c reduces the ubiquitination of c-Jun *in vivo*. RKO cells were transfected with an empty vector or vector encoding KDM5c-dGFP. After immunoprecipitation of c-Jun from lysates, the levels of c-Jun and ubiquitinated c-Jun were determined by Western blot using antibodies against c-Jun or Ub. **(B)** Re-immunoprecipitation of the ubiquitinated c-Jun. After the ubiquitination assay performed as in **(A)**, the proteins were eluted from the beads using SDS sample buffer, re-incubated with antic-c-Jun antibody, and then c-Jun ubiquitination was detected as in **(A)**. **(C)** Time course of c-Jun loss in either RKO or HCT-8 cells. Cells transfected with either the empty vector or KDM5cOE were treated with cycloheximide (CHX) (100 ug/ml) to inhibit protein biosynthesis before examination. The level of β-Actin was detected as the loading control. **(D)** Time course of c-Jun loss in the same cells as in **(C)** after the combined treatment of proteasome inhibitor VELCADE and CHX. Cells were first treated by 100 nM VELCADE for 12 h, then combined with 5 mg/ml CHX for another 2 h. β-Actin was detected as the loading control. **(E,F)** Quantitation of Western blots. Bands corresponding to c-Jun got from RKO **(E)** and HCT-8 **(F)** transfected with an empty vector or vector encoding KDM5c-dGFP and treated with CHX or CHX and VELCADE were quantified. C-JUN levels were normalized toβ-Actin. Data represent mean ± SD from two biological replicates. Non-significant, *P* > 0.05; *P ≤ 0.05; **P ≤ 0.01.

Since FBXW7 is a ubiquitin ligase that targets c-Jun for proteasome-mediated degradation, we examined whether KDM5c overexpression decreases c-Jun ubiquitination by suppressing FBXW7 expression. Immuno-purified c-Jun was incubated with crude lysates of RKO cells transfected with wild-type KDM5c. Consistent with previous report, FBXW7-promoted formation of Ub-c-Jun conjugates, which were immunoprecipitated with anti-c-Jun antibody and detected using an anti-ubiquitin antibody. Notably, in the c-Jun immunoprecipitates, the predominant ubiquitylated band was detected above 37 kDa (Figure 5A). The KDM5c-OE group exhibited lower ubiquitin level and higher c-Jun protein level. The putative ubiquitin-c-Jun conjugates were eluted from the beads using SDS sample buffer and re-immunoprecipitated with the anti-c-Jun antibody, indicating Ub is covalently conjugated to c-Jun (Figure 5B). To examine whether KDM5c overexpression can slow c-Jun metabolism, RKO cells were treated with CHX (1 μM) to inhibit protein synthesis, and then c-Jun protein dynamics were measured by western blot. The c-Jun protein level decreased gradually within the 2 h after CHX treatment (Figure 5C) and KDM5c-OE cells exhibited obviously reduced c-Jun degradation rate after CHX treatment. Moreover, the highly specific proteasome inhibitor VELCADE inhibited c-Jun degradation (Figure 5D), suggesting that the observed decrease in c-Jun content is largely due to degradation by the 26S proteasome. With combined treatment of CHX and VELCADE, KDM5c-OE cells further extended the c-Jun metabolic time. Interestingly, when colon cells were treated with both CHX and VELCADE, there was less of a difference of metabolic rate between KDM5c-OE colon cancer cells and control cells than that with CHX treatment alone. These data are statistically significant (Figures 5E,F) and suggest that KDM5c can inhibit the ubiquitin-26S proteasome degradation pathway of c-Jun.

### KDM5c Promotes Cell Proliferation by Downregulating FBXW7 but Upregulating c-Jun in Colon Cancer Cells

**FIGURE 6:**
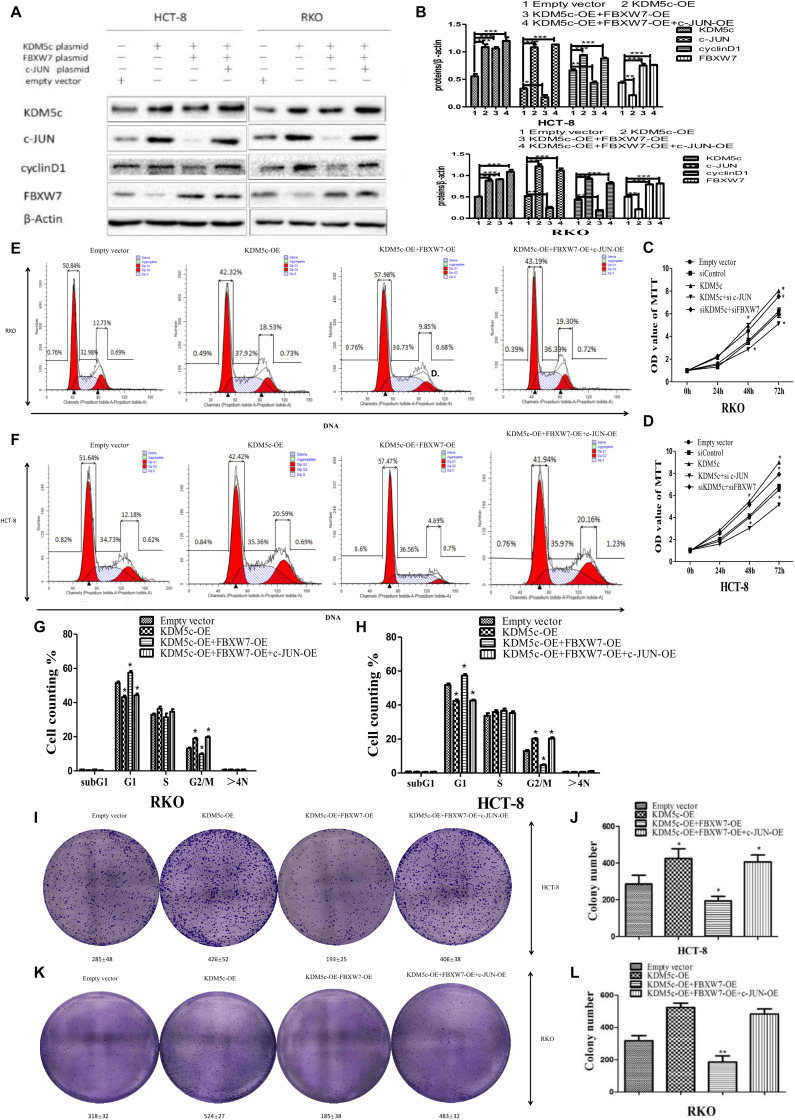
Overexpression of KDM5c promotes colon cancer cells by downregulating FBXW7 to indirectly change levels of the downstream regulator c-Jun. **(A,B)** Representative Western blots **(A)** and quantification **(B)** showing the protein levels of c-Jun, cyclin D1, and FBXW7 in either HCT-8 or RKO cells transfected by empty vector, KDM5c-OE, KDM5c-OE/FBXW7-OE or KDM5c-OE/FBXW7-OE/c-Jun-OE. **(C,D)** MTT assays to detect cell proliferation rates of the cell lines described in **(A)** 72 h after transfection. **(E,F)** Representative flow plots of the same cell lines as in **(A)**. **(G,H)** Statistical results showing that the fraction of G2/M phase cells was significantly increased in KDM5c-OE and KDM5c-OE + FBXW7-OE + c-Jun-OE and decreased in KDM5c-OE + FBXW7-OE RKO (left) and HCT-8 (right) cells. **(I)** 2-D colony formation assay showing colony formation changes in HCT-8 cells transfected by empty vector, KDM5c-OE, KDM5c-OE/FBXW7-OE, or KDM5c-OE/FBXW7-OE/c-Jun-OE. **(J)** In HCT-8 cells KDM5c-OE significantly increased colony formation compared to empty vector, and KDM5c-OE/FBXW7-OE significantly decreased colony formation compared to KDM5c-OE/FBXW7-OE/c-Jun-OE line. **(K)** 2-D colony formation assay showing colony formation changes in RKO cells transfected by empty vector, KDM5c-OE, KDM5c-OE/FBXW7-OE or KDM5c-OE/FBXW7-OE/c-Jun-OE. **(L)** In RKO cells, the KDM5c-OE group exhibited significantly increased colony formation compared to empty vector, while KDM5c-OE/FBXW7-OE exhibited significantly decreased colony formation compared to KDM5c-OE/FBXW7-OE/c-Jun-OE line. Column: mean; Error bar: standard error of the mean (SEM). Statistical analysis was performed and *p*-values were calculated. * Above the bars indicates significant difference; *P ≤ 0.05; **P ≤ 0.01; ***P ≤ 0.001.

The results described above suggest a mechanism in which KDM5c promotes cell proliferation by downregulating FBXW7 but upregulating c-Jun. To test this hypothesis, we next performed functional studies to measure cell proliferation and cell cycle progression. We co-transfected wild type KDM5c and FBXW7 as the KDM5c-OE/FBXW7-OE group (Figures 1A,B), which inhibited growth of RKO/HCT-8 cells (Figures 6C,D) and reduced colony formation (Figures 6I–L), with fewer G2/M phase cells and more G2 phase cells (Figures 6E–H). FBXW7 overexpression eliminated the oncogenicity of KDM5c, indicating downregulation of FBXW7 is necessary for tumorigenesis of KDM5c. However, colon cancer cells co-transfected with KDM5c, FBXW7, and c-Jun as KDM5c-OE/FBXW7-OE/c-Jun-OE cells, exhibited significantly increased cell growth and increased colony formation, with more G2/M phase cells and fewer G1 phase cells. The results show that C-JUN overexpression improves cell proliferation after FBXW7 overexpression, indicating that c-Jun is the downstream target of FBXW7.

### TCGA Database Show High Expression of KDM5c in Colon Cancer Tissue Consistent With High Methylation in KDM5c DNA and Poor Overall Survival

**FIGURE 7:**
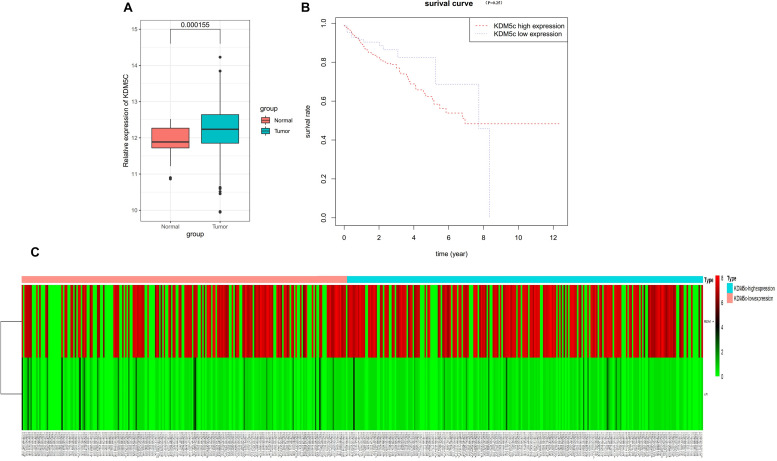
TCGA database analysis. **(A)** TCGA dataset analysis revealed that the KDM5c mRNA levels were significantly increased in colon cancer, (*P* < 0.05). **(B)** Overall survival rates of patients with colon cancer stratified by the expression of KDM5c were analyzed via Kaplan-Meier survival analysis. **(C)** Heat map of the methylation levels of the FBXW7 and JUN genes in 473 human colon cancer tissues as sourced from TCGA. The upper panel shows that colon cancer tissues with higher KDM5c expression exhibit higher methylation levels in the FBXW7 gene. The lower panel shows that KDM5c expression in colon cancer tissue has no relationship with the methylation level in the c-Jun gene.

To assess KDM5c expression levels in colon cancer, we first analyzed the expression of KDM5c mRNA in 464 colon cancer tissues samples from a TCGA dataset (The Cancer Genome Atlas)^4^ and found that KDM5c expression was significantly increased in colon cancer tissue samples compared with normal tissue samples (Figure 7A). Patients with high expression of KDM5c exhibited decreased overall survival rates compared to those with low expression of KDM5c in the first 7 years, *p* = 0.25 (Figure 7B). The TCGA database DNA methylation analysis showed that higher methylation of FBXW7 DNA in colon cancer tissue correlated with high expression of KDM5c, (*p* < 0.05) but there was no association of JUN DNA methylation with KDM5c expression (*p* > 0.05) (Figure 6C). There were no direct differences for gender, pathological type, and tumor stage between the high and low KDM5c expression groups. The clinical information for all samples is provided in the Supplementary Material.

## Discussion

Colorectal cancer (CRC) is one of the leading causes of cancer death, but effective treatments remain limited, leading to a critical demand to identify and exploit novel therapeutic target. In this study, we identified the histone demethylase KDM5c as an interesting target candidate. KDM5c plays an important role in controlling human colon cancer cell proliferation. Specifically, KDM5c protein level can alter colon cancer cell growth by deregulating transcription of the cancer cell repressor gene FBXW7 (Figure 2), thereby, modulating c-Jun degradation via the ubiquitin-proteasome pathway (Figure 4). Thus, our work has revealed a novel function of KDM5c and the underlying mechanism of this function.

Colorectal cancer is a complex disease and was originally thought to result from genetic alterations in key regulatory genes and pathways (24). Later discoveries revealed that epigenetic modifications such as DNA methylation, histone modifications, and non-coding RNA play more essential roles in CRC pathogenesis (25). However, the relationship between these genetic and epigenetic contributions remains to be clarified. In this study, enhanced KDM5c expression increased c-Jun protein level and knock-down of KDM5c reduced c-Jun protein level (Figure 2), indicating strong association of KDM5c with c-Jun function. Modulation of c-Jun by KDM5c suggests epigenetic mechanisms target key gene regulators during CRC development. However, in a previous study, JARID1C promoted metastasis of breast cancer cells via down regulation of BRMS1 expression, and silencing of JARID1C dramatically increased BRMS1 expression, both at the mRNA and protein level (26). This is opposite to the way KDM5c regulates the c-Jun protein, suggesting different mechanisms may allow different functions of KDM5c in different cancer types. In the future, KDM5c inhibitor development, *in vivo* detection of KDM5c, and animal experiments can be applied to further investigate the importance of KDM5c as a target for CRC treatment.

As an important tumor suppressor by the negative regulation of many oncogenic proteins, FBXW7 is under tight control through various mechanisms, including non-coding RNA, methylation, and other genetic regulation (27). Our work here indicates that FBXW7 is a critical downstream target modulated by KDM5c, indicating a new regulatory mechanism by which FBXW7 is regulated. Whether this regulation is specific to CRC or also exists in other cancer types remains to be determined. As the histone methyltransferase EZH2 catalyzes H3K27me3 on FBXW7 (28), future research should investigate if EZH2 may counteract the action of KDM5c to balance the FBXW7 methylation level.

Histone methylation cooperates with DNA modification to modulate gene expression programming, despite the requirement of these two systems for different sets of enzymes to catalyze different chemical reactions (29). Histone methylation helps to direct DNA methylation patterns, and DNA methylation is facilitated by the DNMT3 binding partner, DNMT3L, which binds to chromatin by recognizing the K4 residue on histone H3 (30). If this histone moiety is methylated, the complex cannot bind and the underlying DNA region is thus protected from *de novo* methylation. Generally, H3K4me3 binds within the TSS region and up to 5 kb downstream from actively transcribed genes (31), but it is unclear how H3K4me3 downstream of transcription region affects DNA methylation in the TSS region. In this work, a H3K4me3 peak interacting with KDM5c is located downstream of the FBXW7 coding sequence, and the CpG island that exhibits increased methylation after KDM5c overexpression is located upstream of the TSS. If the H3K4me3 peak acts as the anchor of DNMT3L and recruits DNMT3 to methylate the CpG island in the TSS region, then the two sites, more than 16000 bp apart. As shown by our 3C-ChIP data, the CpG island in the FBXW7 gene TSS region and a H3K4me3 peak in the downstream portion of the FBXW7 gene are indeed physically close and connected by DNMT3 (Figure 4). Therefore, our results provide insights into the coordination between histone methylation and DNA methylation.

## Conclusion

In conclusion, KDM5c promotes *in vitro* colon cancer cell growth by a mechanism involving demethylation of H3K4me3 in the TSS and downstream of the tumor suppressor gene *FBXW7*. H3K4me3 demethylation may recruit DNMT3b, resulting in methylation of the CpG island located near the TSS. This causes downregulation of *FBXW7* expression, which reduces the ubiquitin-proteasome-mediated degradation of proto-oncogene c-Jun. The *in vivo* data from TCGA validate our conclusions. Our results demonstrate a novel epigenetic regulatory pathway in colon cancer and suggest KDM5c demethylase as an exciting potential target for colon cancer therapy.

## Data Availability Statement

All datasets generated for this study are included in the article/Supplementary Material.

## Ethics Statement

Written informed consent was obtained from the individual(s) for the publication of any potentially identifiable images or data included in this article.

## Author Contributions

HL and BC designed the study. HL and NM developed the methodology, performed the analyses, and collected the data. LZ analyzed the data. HL and GY wrote the first draft. All the authors contributed to the review and revision of the manuscript, and read and approved the final manuscript.

## Conflict of Interest

The authors declare that the research was conducted in the absence of any commercial or financial relationships that could be construed as a potential conflict of interest.

## Footnotes

1 https://portal.gdc.cancer.gov/

2 http://www.urogene.org/methprimer/

3 http://quma.cdb.riken.jp/

4 http://ualcan.path.uab.edu/analysis.html
