# Supplementary material for: KDM5c Promotes Colon Cancer Cell Proliferation Through the FBXW7-c-Jun Regulatory Axis
Source: Front Oncol. 2020 Sep 16;10:535449. doi: 10.3389/fonc.2020.535449 (PMC7526003; doi:10.3389/fonc.2020.535449)
Supplement: Supplementary file 2 [file Table_2.DOCX]

(-1626/-1419)

GGAAAGTACACCTGGTCCTGCCAAATCGCACTCTTATATCCTGGCATCCTATCCAGGCTCTGCGAGGATGGAAACTGCGAGGCAGGGGAGGGAAGCGGGCTGTTTGGCCACCACCTCCCTAGTGCTGCAGGCGACCCTGTCACACTAACTCCTGGCAGCCCAGTGAGGTGGACGGCACCGGCCCCACCTGCAGATGAGGGAAATGAA

(-1440/-1181)

CCTGCAGATGAGGGAAATGAAGCTCGGAGGAGTTCCGTGATTTGCTTGCTTCACACTGTGGTAGCCTGGCCACGAAAGAACCAGGATTCCCGACTTCGGGATTCTTTCCACCACACACTTTCGTCCCTAAGGGGTGGGGGGCGGGGGGAGAATAAAATAACCGCGGAAAAGGAACCACTTACATGTGTCTAGCGCTTCCTAGAGGCTACCCAGGATATGCGCCCACCACCCGGCCGGGAGTGCAGAGATTTGAAGTCCA

(-1202/-935)

AGTGCAGAGATTTGAAGTCCAGGTTCTACCCCGGGCTCCGAGTACTACTGCGTGACTTTATGCGAGTGTCCGCCGCCTTCTGGGCTTGTTTTCCCGGAAGCAACTCGGCGCGGATGGAGTGTGTGTGTGCGCGCGCGCGCGCGTTATGTTGTGCGTGTTGTGTTAAGCGTGTGCGTGTTGTCCGGGGGCGGGAGGGGGAGTAGACTAACACCGGGGTTCCCCGAGTTTCGGATCGCCTACACGCTTGTTCCCATCTGGACCCTGTTA

(-842/-503)

GGTGCAACGGAGACTCAGCTGAGCGTCCAGTTTCGGGCAATACAAATCTCTCGGCTTCTACGAGCAGCCAGACGACCCCGCGGACCGTCGCTCCTGAACTTGACCGAGATGCAAACTTCGGAGTGTTCTCAACGTGGGGGGCCGACTCTCGGGAGACCGCCCCTAAACTTAAGTCCCCTTAGGCTCGCCCCCACCTGGGACTTCACAGAGCCACCTTAAGGGCGGTATTCCCGCCCCCCCGGAAGTGCGGGGGGGTGGCAGCGTACTTGGATTCTCAGCCTCCAGCCCCGCGCGGTGGCGGCCGCCGGTGGATGACTTCGGGCCCCACAAGTGGGGAAA

(-842/-503)

AAAACAACTGGCCAGGTTCCCTGGCCTCCCGGGTCCCTGCATCCCCCGCATCCCCGTCCGCAGCCGTGAACTTGAGCCCCCCTCCATCAGAGGTTGCGAGCGTCCGCCCGCTCGCGGCAGCCACCGTCACTAGACAGTCAAACCCCAAGACGTCAGCCCACAATGCACCGGGCGGGCCGGGAAAAACGGCCCGGGGAGGGGACCGGGGAAGAGAGGGCCGAGAGGCGTGCGGCAGGGGGGAGGGTAGGAGAAAGAAGGGCCCGACTGTAGGAGGGCAGCGGAGCATTACCTCATCCCGTGAGCCTCCGCGGGCCCAGAGAAGAATCTTCTAGGGTGGAGTCTCCATGGTGACGGGCGGGCCCGCCCCCCTGAGAGCGACGCGAGCCAATGGGAAGGCCTTGGGGTGACATCATGGGCTAT
