# Supplementary material for: KDM5c Promotes Colon Cancer Cell Proliferation Through the FBXW7-c-Jun Regulatory Axis
Source: Front Oncol. 2020 Sep 16;10:535449. doi: 10.3389/fonc.2020.535449 (PMC7526003; doi:10.3389/fonc.2020.535449)
Supplement: Supplementary file 4 [file Table_4.DOCX]

FBXW7 CpG 152324318-152326358

ggttgtaaggtggagccactgacaatgaaaacagaagatca**CG**ctgctgaaaaaccctatgtggtacacacttaactggaggtgttctctgctgggctgcagaactctgatgtttgcactcttttccttcaaagcctacaaaggttaaactctctgaggtgtttagcttcacaaaatcccctaaatctgtgtgttttactcctttaatttccaccattcccctgttgtaagataggaagagggggaaaaacaaaaaatatttattttgtagccatgtgagaccatcacttgttggtctgattacctgtgagtcttatttggaaggctgtagcaggggattggtca**CG**taaagacagaaaaatccagggtaatttaagcaggatatttaacctggctcttggaacttcaggtcaagggcagtagggtcaggtgctagtctgtgagtctctccttggttttgccacatcctcctttgcccctctcaccacctttcc**CG**cagcattttaatgaaca**CG**agcccttggatttacccagttaaaagtttccttagtttactgtttgcagatggtagtggcagtagtgggagaaacccagagcttcccacatttgtctataatacacttgctcctaccttaccccctcctccaggtggaaagacttgtaaaatccattactactgcttcccaaggaggtagagtgtactagggtccagggacagaattgtggaatctgctttcatcattgctgctgctttaatctttttctcatactattcactgaagacagatttctgaggattctggtccttttatcttaaatttgttttcctagagaactacactttaaacttcatgagtttctacttaactttcctttccaggataacatctgtaggaaaagctgcataatagtaataacacttattttagaaagaaaataacctttgagtcagacctgaaacaggccaacatttcttgatgacaa**CG**tacactgatgaaccaagaccagaagctct**CG**aaagctccaaac**CG**taagaaagaaccattcgtaaaagtgagggactattaaattcttcccctgacagtcaaaaatccttcagcaatctcccagaggatggctcccttccaatcctcccttcc**CG**c**CG**ccctaacaatctg**CGCG**aggatgggtcccttcccttgct**CG**gcattt**CG**tctccttttccactaaagactcaggttgtgactctgtactcaagc**CG**cacccctgtttcctttcatggctagggggaggggaggaatgtattacaactcatttttacaatgcactgcagtttacaaag**CG**cttgcacttccaccatatcctctgagcttca**CG**agtgcca**CG**tgagagaggcattcattcattcattcattcatcaaacatttgct**CG**gcc**CG**accaca**CG**ttatccaa**CG**ctgggtctcacccca**CG**cca**CG**ggaagggtgggg**CG**ctctctccctggagcaggtgcagatcaggctgtagggtaactcct**CG**atcagtc**CG**gctttt**CG**agggtctgtttggggccaaatgcctcacctt**CG**ggtggtg**CG**cgcagctgcctgaaga**CGCG**gcctgggcctcctccttcctcccaaaggagcagtccccacctgccc**CG**aaggaaac**CG**ctacagaccaagctctccaggccgccct**CG**ggtcaggggtaagat**CG**gg**CG**gcagcttaggct**CG**act**CG**gctcctccccttcctctcttcagttctga**CGCG**gc**CG**tagagg**CG**gcag**CG**gcggtgg**CG**gctg**CG**agtcc**CGCG**gcc**CG**agg**CG**cacccacc**CG**ct**CG**c**CG**atccccacttcctcttcccttggag**CG**tcatcagag**CGCG**ccagagg**CG**cccaggg**CG**gggtggctggaagggggaaaaaggccag**CG**gaggaagcctcagag**CG**gaaagaagagggagggggaagagagcggttgctaggtgacttg**CG**tcat**CG**gcagg**CG**c**CG**ctctcctcc**CG**cccctctctctggagtgagg**CG**agagccc**CG**cacaga
