# Supplementary material for: KDM5c Promotes Colon Cancer Cell Proliferation Through the FBXW7-c-Jun Regulatory Axis
Source: Front Oncol. 2020 Sep 16;10:535449. doi: 10.3389/fonc.2020.535449 (PMC7526003; doi:10.3389/fonc.2020.535449)
Supplement: Supplementary file 5 [file Table_5.DOCX]

JUN 58778791-58786047

TCTTGTCATCACTGTATACCTACCTACCCCACCCCCTCCCCAGCTCAGTGCCTGGCTCACAGTAGGCTTTCAGTTACCCTCTGCAGATCAGTGAAAGCTAGGTGAGTGCCCGGAGTGAAGAAAAGTTGGCAGGTTTCCCACTGATACCAGCTGCTGTTGGTTTCTGAACACTCAAAGCCGCAAATACCTTAGGGCTGGGGGCAATGAACCCAAGGCTGAATTCCAAGTTCAGAAGCAGCGAAGTCTGAATTTAGAACCTAGGAACTTAAACTGCTGCAGGTCCAACTTCAAGCCCCAGTTTTAGACAGAGGCTTGGGAAAGATCTGACTTCTAACCCGGTTCCCCCTCCCCTCCTCCCCTCGATGCTTCTCACAGGAAAGTACACCTGGTCCTGCCAAATCGCACTCTTATATCCTGGCATCCTATCCAGGCTCTGCGAGGATGGAAACTGCGAGGCAGGGGAGGGAAGCGGGCTGTTTGGCCACCACCTCCCTAGTGCTGCAGGCGACCCTGTCACACTAACTCCTGGCAGCCCAGTGAGGTGGACGGCACCGGCCCCACCTGCAGATGAGGGAAATGAAGCTCGGAGGAGTTCCGTGATTTGCTTGCTTCACACTGTGGTAGCCTGGCCACGAAAGAACCAGGATTCCCGACTTCGGGATTCTTTCCACCACACACTTTCGTCCCTAAGGGGTGGGGGGCGGGGGGAGAATAAAATAACCGCGGAAAAGGAACCACTTACATGTGTCTAGCGCTTCCTAGAGGCTACCCAGGATATGCGCCCACCACCCGGCCGGGAGTGCAGAGATTTGAAGTCCAGGTTCTACCCCGGGCTCCGAGTACTACTGCGTGACTTTATGCGAGTGTCCGCCGCCTTCTGGGCTTGTTTTCCCGGAAGCAACTCGGCGCGGATGGAGTGTGTGTGTGCGCGCGCGCGCGCGTTATGTTGTGCGTGTTGTGTTAAGCGTGTGCGTGTTGTCCGGGGGCGGGAGGGGGAGTAGACTAACACCGGGGTTCCCCGAGTTTCGGATCGCCTACACGCTTGTTCCCATCTGGACCCTGTTACCCACCAATTGCGCCCACTATAAAAACTGCCCCTCCGAGGCAAAGCTGTGAACCCCCGCGCCCTTTCCCCCACGGTCCCGGAGGATGAAGTGGGGTGCAACGGAGACTCAGCTGAGCGTCCAGTTTCGGGCAATACAAATCTCTCGGCTTCTACGAGCAGCCAGACGACCCCGCGGACCGTCGCTCCTGAACTTGACCGAGATGCAAACTTCGGAGTGTTCTCAACGTGGGGGGCCGACTCTCGGGAGACCGCCCCTAAACTTAAGTCCCCTTAGGCTCGCCCCCACCTGGGACTTCACAGAGCCACCTTAAGGGCGGTATTCCCGCCCCCCCGGAAGTGCGGGGGGGTGGCAGCGTACTTGGATTCTCAGCCTCCAGCCCCGCGCGGTGGCGGCCGCCGGTGGATGACTTCGGGCCCCACAAGTGGGGAAACAACAACCACCCCTCGCCCGCACCCCTGGCCCAAAACAACTGGCCAGGTTCCCTGGCCTCCCGGGTCCCTGCATCCCCCGCATCCCCGTCCGCAGCCGTGAACTTGAGCCCCCCTCCATCAGAGGTTGCGAGCGTCCGCCCGCTCGCGGCAGCCACCGTCACTAGACAGTCAAACCCCAAGACGTCAGCCCACAATGCACCGGGCGGGCCGGGAAAAACGGCCCGGGGAGGGGACCGGGGAAGAGAGGGCCGAGAGGCGTGCGGCAGGGGGGAGGGTAGGAGAAAGAAGGGCCCGACTGTAGGAGGGCAGCGGAGCATTACCTCATCCCGTGAGCCTCCGCGGGCCCAGAGAAGAATCTTCTAGGGTGGAGTCTCCATGGTGACGGGCGGGCCCGCCCCCCTGAGAGCGACGCGAGCCAATGGGAAGGCCTTGGGGTGACATCATGGGCTATTTTTAGGGGTTGACTGGTAGCAGATAAGTGTTGAGCTCGGGCTGGATAAGGGCTCAGAGTTGCACTGAGTGTGGCTGAAGCAGCGAGGCGGGAGTGGAGGTGCGCGGAGTCAGGCAGACAGACAGACACAGCCAGCCAGCCAGGTCGGCAGTATAGTCCGAACTGCAAATCTTATTTTCTTTTCACCTTCTCTCTAACTGCCCAGAGCTAGCGCCTGTGGCTCCCGGGCTGGTGTTTCGGGAGTGTCCAGAGAGCCTGGTCTCCAGCCGCCCCCGGGAGGAGAGCCCTGCTGCCCAGGCGCTGTTGACAGCGGCGGAAAGCAGCGGTACCCACGCGCCCGCCGGGGGAAGTCGGCGAGCGGCTGCAGCAGCAAAGAACTTTCCCGGCTGGGAGGACCGGAGACAAGTGGCAGAGTCCCGGAGCCAACTTTTGCAAGCCTTTCCTGCGTCTTAGGCTTCTCCACGGCGGTAAAGACCAGAAGGCGGCGGAGAGCCACGCAAGAGAAGAAGGACGTGCGCTCAGCTTCGCTCGCACCGGTTGTTGAACTTGGGCGAGCGCGAGCCGCGGCTGCCGGGCGCCCCCTCCCCCTAGCAGCGGAGGAGGGGACAAGTCGTCGGAGTCCGGGCGGCCAAGACCCGCCGCCGGCCGGCCACTGCAGGGTCCGCACTGATCCGCTCCGCGGGGAGAGCCGCTGCTCTGGGAAGTGAGTTCGCCTGCGGACTCCGAGGAACCGCTGCGCACGAAGAGCGCTCAGTGAGTGACCGCGACTTTTCAAAGCCGGGTAGCGCGCGCGAGTCGACAAGTAAGAGTGCGGGAGGCATCTTAATTAACCCTGCGCTCCCTGGAGCGAGCTGGTGAGGAGGGCGCAGCGGGGACGACAGCCAGCGGGTGCGTGCGCTCTTAGAGAAACTTTCCCTGTCAAAGGCTCCGGGGGGCGCGGGTGTCCCCCGCTTGCCACAGCCCTGTTGCGGCCCCGAAACTTGTGCGCGCAGCCCAAACTAACCTCACGTGAAGTGACGGACTGTTCTATGACTGCAAAGATGGAAACGACCTTCTATGACGATGCCCTCAACGCCTCGTTCCTCCCGTCCGAGAGCGGACCTTATGGCTACAGTAACCCCAAGATCCTGAAACAGAGCATGACCCTGAACCTGGCCGACCCAGTGGGGAGCCTGAAGCCGCACCTCCGCGCCAAGAACTCGGACCTCCTCACCTCGCCCGACGTGGGGCTGCTCAAGCTGGCGTCGCCCGAGCTGGAGCGCCTGATAATCCAGTCCAGCAACGGGCACATCACCACCACGCCGACCCCCACCCAGTTCCTGTGCCCCAAGAACGTGACAGATGAGCAGGAGGGCTTCGCCGAGGGCTTCGTGCGCGCCCTGGCCGAACTGCACAGCCAGAACACGCTGCCCAGCGTCACGTCGGCGGCGCAGCCGGTCAACGGGGCAGGCATGGTGGCTCCCGCGGTAGCCTCGGTGGCAGGGGGCAGCGGCAGCGGCGGCTTCAGCGCCAGCCTGCACAGCGAGCCGCCGGTCTACGCAAACCTCAGCAACTTCAACCCAGGCGCGCTGAGCAGCGGCGGCGGGGCGCCCTCCTACGGCGCGGCCGGCCTGGCCTTTCCCGCGCAACCCCAGCAGCAGCAGCAGCCGCCGCACCACCTGCCCCAGCAGATGCCCGTGCAGCACCCGCGGCTGCAGGCCCTGAAGGAGGAGCCTCAGACAGTGCCCGAGATGCCCGGCGAGACACCGCCCCTGTCCCCCATCGACATGGAGTCCCAGGAGCGGATCAAGGCGGAGAGGAAGCGCATGAGGAACCGCATCGCTGCCTCCAAGTGCCGAAAAAGGAAGCTGGAGAGAATCGCCCGGCTGGAGGAAAAAGTGAAAACCTTGAAAGCTCAGAACTCGGAGCTGGCGTCCACGGCCAACATGCTCAGGGAACAGGTGGCACAGCTTAAACAGAAAGTCATGAACCACGTTAACAGTGGGTGCCAACTCATGCTAACGCAGCAGTTGCAAACATTTTGAAGAGAGACCGTCGGGGGCTGAGGGGCAACGAAGAAAAAAAATAACACAGAGAGACAGACTTGAGAACTTGACAAGTTGCGACGGAGAGAAAAAAGAAGTGTCCGAGAACTAAAGCCAAGGGTATCCAAGTTGGACTGGGTTGCGTCCTGACGGCGCCCCCAGTGTGCACGAGTGGGAAGGACTTGGCGCGCCCTCCCTTGGCGTGGAGCCAGGGAGCGGCCGCCTGCGGGCTGCCCCGCTTTGCGGACGGGCTGTCCCCGCGCGAACGGAACGTTGGACTTTTCGTTAACATTGACCAAGAACTGCATGGACCTAACATTCGATCTCATTCAGTATTAAAGGGGGGAGGGGGAGGGGGTTACAAACTGCAATAGAGACTGTAGATTGCTTCTGTAGTACTCCTTAAGAACACAAAGCGGGGGGAGGGTTGGGGAGGGGCGGCAGGAGGGAGGTTTGTGAGAGCGAGGCTGAGCCTACAGATGAACTCTTTCTGGCCTGCCTTCGTTAACTGTGTATGTACATATATATATTTTTTAATTTGATGAAAGCTGATTACTGTCAATAAACAGCTTCATGCCTTTGTAAGTTATTTCTTGTTTGTTTGTTTGGGTATCCTGCCCAGTGTTGTTTGTAAATAAGAGATTTGGAGCACTCTGAGTTTACCATTTGTAATAAAGTATATAATTTTTTTATGTTTTGTTTCTGAAAATTCCAGAAAGGATATTTAAGAAAATACAATAAACTATTGGAAAGTACTCCCCTAACCTCTTTTCTGCATCATCTGTAGATACTAGCTATCTAGGTGGAGTTGAAAGAGTTAAGAATGTCGATTAAAATCACTCTCAGTGCTTCTTACTATTAAGCAGTAAAAACTGTTCTCTATTAGACTTTAGAAATAAATGTACCTGATGTACCTGATGCTATGGTCAGGTTATACTCCTCCTCCCCCAGCTATCTATATGGAATTGCTTACCAAAGGATAGTGCGATGTTTCAGGAGGCTGGAGGAAGGGGGGTTGCAGTGGAGAGGGACAGCCCACTGAGAAGTCAAACATTTCAAAGTTTGGATTGTATCAAGTGGCATGTGCTGTGACCATTTATAATGTTAGTAGAAATTTTACAATAGGTGCTTATTCTCAAAGCAGGAATTGGTGGCAGATTTTACAAAAGATGTATCCTTCCAATTTGGAATCTTCTCTTTGACAATTCCTAGATAAAAAGATGGCCTTTGCTTATGAATATTTATAACAGCATTCTTGTCACAATAAATGTATTCAAATACCAATAACAGATCTTGAATTGCTTCCCTTTACTACTTTTTTCTTCCCAAGTTATATACTGAAGTTTTTATTTTTAGTTGCTGAGGTTAACATTGCTGCAATCTGAAGTGATTTGAAAATTAAGAAGCAAAGAATTTAGGAAGAGTCAAACTAGAAGTTGTGATCTGAATGAAACAGTCTCCCAATTTCATTAATTCTTTCCCTCTTTCTCCATCTCTATCTCTCTCTCTACTACCTCACCCCCAAATAAACCCCTATGCCTATGGAAACTAACAGTTTAGCATTTTGGGTCTGTCTGTAGCTTATTTCTTAATTTGTTTCTAAAATAATCAACCTTGTTTAAATATAGAATAAGTCTCAAAAGATAGTAATCTAATACTATGGAAAAGATTCACAAGAGAAAACATGAACTAGTACTAACTCAATGATTACATGCGTAAATCAAACCTCAGCTCTGGGGAAATGGGAAACTTGAATTCTACCTACAGATATAACTTAGGAGATCAACAAGGCAGTGCTTAACAAGGGAACAATTTTTTGCTTGGTTGCTTTTCATACCAAGAGCCAGTGTGATTATCAGGCAGCAAAAGCAATTTGATATCCAGGAATATTTCACAAACCACTAATTGAGTCAGGGCTGTTTGCTCATTAGCAGTGTTCTAGTACCCAGTAGGTCTGGGAGTGCTAATATCCTGCCTGTGGCTGTAAATCCAAATACACAAAGAGTTTCTCCTTAGGAGTATATCTAGGAGTGGAAGAGATTGGACAGGCTTGTTAGCTTCGGATCCTTTGTAATAACACTTCTTGAACAAAGGGTTACTGGGAGCAGAGCCCAGTTTGCCAAACTGAATTTGCCGATGTCAGCAACCCGGAAGGTGAGATGATTTTGACCATATGTAGAAATAGCAGAGGGACAATAAAGCATTTTGGGGTGGGGCTCTGTGGAAGCTAAACAAGAGCCAACTAGAAAAAATAAGCAGGCTGGGGAATTAATTACCTGCAAAGGTAGACTAAGCCCAGACAACAGTACAGCACAGAAAAGCCACACCACTCCCCAGTTTGCTTTAGATTTCAGTGTTAAAAAAAAAAAAAGTCTGAAACTCAGGCCTATCCCCATGCTTGATTGTTTCAATTAACATTCTAAATGTATATTCTTGAAATTCTAACCATTCTAACCAAAAGGGGTAGAAAAAAGTAATTGTGGTAGAGGAAATTAACCCATGATCTCTGGTAAATTTTGGAGACAAAAGATTAACTGCTTATAAACACTTAATGGTTAAGCTTATTATGGGCCTTCAATGAGTTAATCTTTGAATTAAGGTAACACTAATAGTTGGAGAAGATGAAGCTGGTGACACATCGTCATTAATCCTCTTAATTTTTTTTACACATATATATATGTTACATATATAAAATAATTCAATCAAGGACATTTAACCCTCTCTGTCTATCAAGACAGAGGGTTCATGAACTCAGTGAAAATGCCCAGAAAGTTGTGTTTGTGTGTCTGTACACACTCTTCTGAGGAGAGAAATTTTTGCTTTTATTCGACCCTCCCAGAGTTCCATGACTCAAAAAAAAGGGTTAATAAACCATGAACTGACCAGCTACATGCCCACTATCAGAAACATATCATTGTATGGATCTAAATCCCATGCTCTTTCACCCCTTCATGTTTCTGCTTGCATGTATTTGCCTGGAATACCTTTCCTTCTAGTTCAACTGATAAATCCTACCCATTCTTCAAGTCTTGGCGTAAGTGACACCTTCTCTGTAAAGCTTCCCTTAGTAACTCCATCTTCAAAAGGAATGAATAATTCCCTAATGAGCATGATAACATTTTATCAAAAATCCTATGATAGCACATACAAAAATGTATTATGAACCTCTGTGTCTCCTGGTCAGAACCCTATCTTCTTCATCAATGGACTCCCAAACTTTTAATGTAGCGCCTGGCATATAGTAGATGTTCATAAAATAAATGTTTGATGACTGAACGAACAAG
